# Determination of the effect of collars containing 10% w/w imidacloprid and 4.5% w/w flumethrin (Seresto®) on the incidence of *Leishmania* and other canine vector-borne pathogen infections in Greece

**DOI:** 10.1186/s13071-023-05678-4

**Published:** 2023-03-07

**Authors:** Panagiota Ligda, Manuela Gizzarelli, Despoina Kostopoulou, Valentina Foglia Manzillo, Anastasios Saratsis, Katerina Saratsi, Susan Michler, Hannah Ringeisen, Annette Boegel, Bettina Schunack, Matthias Pollmeier, Michalis Kontrafouris, Ourania Tsatsaki, Gaetano Oliva, Smaragda Sotiraki

**Affiliations:** 1 Veterinary Research Institute, Hellenic Agricultural Organization–Demeter, 57001 Thermi, Thessaloniki Greece; 2 Department of Veterinary Medicine and Animal Production, University of Naples Federico II, 80137 Naples, Italy; 3 Elanco Animal Health, 40789 Monheim, Germany; 4 Veterinary Centre of Leros, 85400 Platanos, Leros Greece; 5 Vet Clinic, Thoukididou 6, 71307 Heraklion, Crete Greece

**Keywords:** Canine vector-borne pathogens, Imidacloprid, Flumethrin, Collar, Prevention

## Abstract

**Background:**

The objective of this field study was to assess the effect of treating a considerable portion of a dog population naturally exposed to canine vector-borne pathogens (CVBPs) in endemic areas with a 10% w/w imidacloprid/4.5% w/w flumethrin collar (Seresto®) on the transmission of CVBPs and the resulting incidence of infection.

**Methods:**

A total of 479 dogs from two sites were enrolled in the study. Collars were placed on all dogs continuously for 21 months, with replacement of the collar every 7 months. All dogs were examined, including body weight and blood/conjunctival swab collections, every 7 months. Serum samples were analysed for the presence of antibodies against *Leishmania infantum*, *Ehrlichia canis* and *Anaplasma phagocytophilum*. PCR assays were also performed on blood samples and conjunctival swab collected from the dogs for the presence of *L. infantum*, and on blood samples only for the presence of *Ehrlichia* spp. and *Anaplasma* spp. Sand flies were collected, identified to species level and molecularly tested for *L. infantum* throughout two vector activity seasons.

**Results:**

The results showed that the Seresto collar was safe with continuous use. At study inclusion, 419, 370 and 453 dogs tested negative for *L. infantum*, *Ehrlichia* spp. and *Anaplasma* spp., respectively (353 dogs tested negative for any pathogen). Overall, 90.2% of the dogs were protected from *L. infantum* infection on both sites combined. The entomological survey confirmed the presence of competent vectors of *L. infantum* at all monitored locations, namely the sand flies *Phlebotomus neglectus* and *Phlebotomus tobbi*, both of which are regarded as the most important competent vectors in the Mediterranean basin. All captured sand flies tested negative for *L. infantum.* Protection against ticks and fleas was high, with only two dogs showing a low number of ticks and seven dogs having low numbers of fleas at single evaluation time points. Across the entire study population, a number of dogs became infected with tick-transmitted pathogens, but prevention of transmission was 93% for *E. canis* and 87.2% for *Anaplasma* spp. when all cases from both sites were combined.

**Conclusions:**

The Seresto^®^ (10% w/w imidacloprid/4.5% w/w flumethrin) collar significantly reduced the risk of CVBP transmission when compared to previously observed incidences of CVBP infections in two highly endemic areas under field conditions.

**Graphical Abstract:**

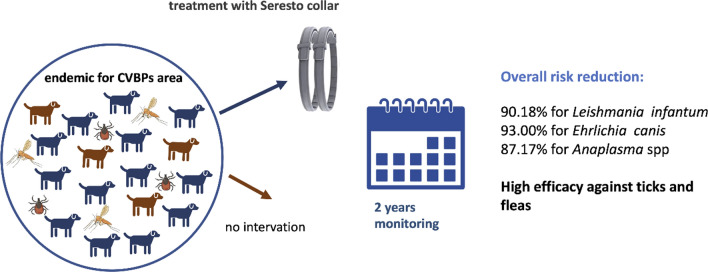

**Supplementary Information:**

The online version contains supplementary material available at 10.1186/s13071-023-05678-4.

## Background

Canine vector-borne pathogens (CVBPs) represent a growing global threat, posing unprecedented challenges to veterinarians especially in endemic areas [1]. During the last decade CVBPs have continued to spread far from their traditional geographical and temporal restraints due to changes in both climatic conditions and pet dog travel patterns, thereby exposing new populations to previously unknown infectious agents [2].

Greece is located in the Mediterranean basin with favourable environmental conditions for pathogen-transmitting vectors and poorly managed stray dog populations, and numerous studies and publications have reported the country to be a highly endemic area for various CVBPs [3]. In such an environment, the availability of effective tools for controlling exposure of dogs and cats to ectoparasites, and thereby to the pathogens they transmit, is crucial to reducing the risk of infection in animals and eventually in humans, considering the zoonotic potential of some CVBPs. Such tools become even more relevant given that Greece also represents a country from which dogs are commonly relocated, either within its own territory or to different countries. Dog relocation can cause dissemination of pathogen and vector populations [4]. All of the above apply at large to *Leishmania infantum*, the causative agent of human and canine leishmaniosis, which is one of the world’s most important zoonotic parasites, with a continuous spread from the Mediterranean areas to continental Europe [5, 6]. Most infected individuals remain asymptomatic, but the role of such asymptomatic individuals as ‘parasite carriers’ is still under careful consideration [7, 8].

The objective of this field study was to assess the effect of treating a dog population naturally exposed to CVBPs within a geographically defined endemic area with a commercially available 10% w/w imidacloprid/4.5% w/w flumethrin collar (Seresto®; Elanco Animal Health, Indianapolis, IN, USA) on the transmission of *Leishmania* and other CVBPs and the resulting incidence of infection.

## Methods

### Study location

**Fig. 1:**
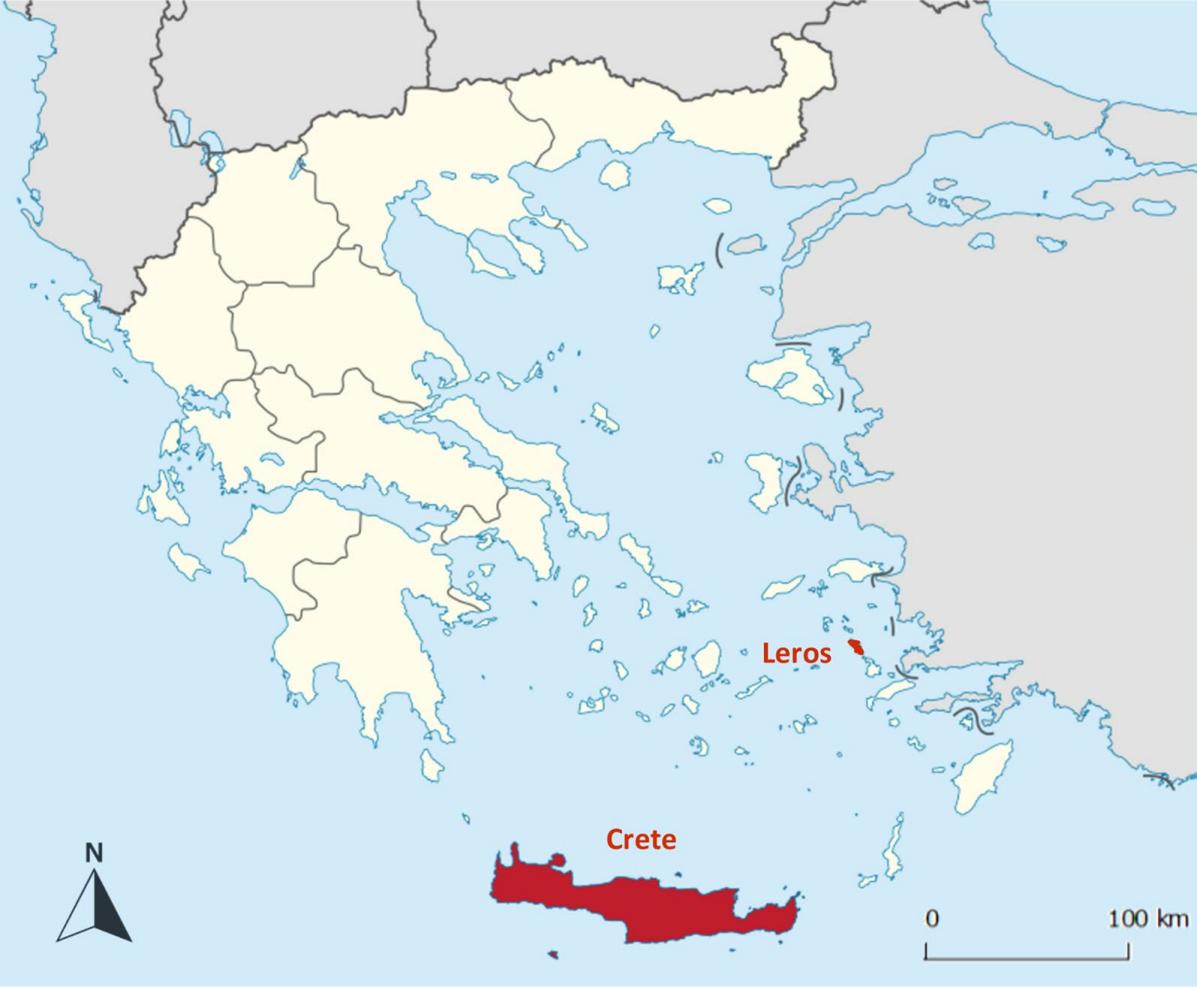
Map of Greece showing the location of Crete and Leros, the two sampling areas

The study was conducted as a field trial in dogs considered highly likely to be naturally exposed to CVBPs at two locations: the Greek islands of Crete and Leros (Fig. 1). These two locations are characterised by contrasting landscapes and demographic and socioeconomic conditions. A previous screening survey confirmed that CVBPs are endemic at these locations [3]. One site was located in the north-eastern part of Crete with a catchment area around the city of Heraklion, and the second site was located on Leros with a catchment area covering the entire island.

### Animals

Inclusion criteria for a dog to participate in the study were that the dog (i) be healthy (normal body condition, normal attitude/behaviour, no visible signs of systemic disease) and non-fractious, so as to allow handling and blood specimen collection; (ii) be at least 7 weeks of age or older; (iii) be free of any skin lesions around their neck; (iv) have not been treated with a long-acting acaricide/insecticide within the efficacy period of these products before the start of the trial; and (v) have no history of hypersensitivity to any of the active substances or excipients of the collar under investigation.

Written owner consent was obtained, and a description of housing conditions was recorded. Food and water were provided as usual by the owner, with no diet restrictions. Each dog’s attending veterinarian and the authors took full responsibility throughout the duration of the study for the welfare and health status of the animals.

### Study design

This study was designed as a single-group multicentre good clinical practice efficacy study and therefore no blinding or randomisation took place. All animals belonged to one group and all received the Seresto® collar for dogs (10% w/w imidacloprid/4.5% w/w flumethrin collar).

Treatment started in May (study day (SD) 0), and dogs continuously wore collars during 2 years of follow-up. Dogs received a collar on SD 0, placed on each dog by the participating veterinarians, and the collar was replaced approximately every 7 months (between SD 205 and 214 [referred as SD 210] and between SD 408 and 428 [referred as SD 420]). In case of loss or destruction of the collar or changes in the dog weight class (applicable to dogs that during growth increased in body weight [BW] from ≤ 8 to > 8 kg), the collar was immediately replaced within 48 h. Any dogs added to the same household during the first year of the study were also included in the study and received a collar.

On SD 0, 210, 420 and 630 (the latter referring to the end of the study period) dogs underwent a physical examination, were weighed on a verified scale and blood was collected. Only dogs in sufficiently good health, as assessed by the veterinarian during the examination, continued to participate in the study. As the study was considered to be a field study, the veterinary examination focused on visible and palpable clinical signs that were documented. Specifically, for the blood samples, approximately 5 ml was collected from each individual animal; the sample was then equally divided into a gel and clot activator (left 30 min at room temperature) and an EDTA tube (slowly tilted at least 5 times), following which the samples were immediately placed in a refrigerator and maintained at 4 °C; they were examined within 2 days of collection. Conjunctival swabs were collected using sterile cotton swabs, manufactured for bacteriological isolation studies, on SDs 0 and 630. One swab per eye was collected by rubbing the swab against the surface of the lower eyelid to collect exfoliating cells. Conjunctival swabs were kept in sterile tubes and stored frozen at − 20 °C until analysed.

### Ectoparasite load

During all physical examinations, the presence and level of tick and flea infestation were recorded. The level of infestation was classified separately for ticks and fleas as negative (0 ticks/fleas), low (1–3 ticks/1–5 fleas), medium (4–10 ticks/6–20 fleas) or high (> 10 ticks/> 20 fleas). A sample of ticks (up to 10 ticks) and fleas (up to 20 fleas) was collected from infested dogs and preserved in 70% ethanol for future morphological identification using taxonomic keys [9, 10].

### Sand fly trapping

Sand flies were collected starting on SD 0 in both locations. Follow-up collections in Heraklion (Crete) were performed between SDs 78 and 80, SDs 134 and 136, SDs 318 and 320, SDs 409 and 411, SDs 533 and SD 534 and SD 661 and 663. Follow-up collections in Leros were performed between SDs 77 and 80, SDs 144 and 146, SDs 338 and 340, SDs 424 and 426, SDs 539 and 542 and SDs 675 and 678. The US Center for Disease Control (CDC) miniature light traps and mechanical aspirators were used for all collections. The light traps were placed close to animal-inhabited biotopes. The traps operated overnight (for 2–4 consecutive nights each time), and the sand flies collected were kept either dry or in 70% ethanol until examination.

All captured sand flies were counted. For species identification, the head and the terminal part of the abdomen of all collected female sand flies were mounted on permanent microscopy slides and subsequently identified using the appropriate keys based on the morphology of the pharynx and the genitalia armature and documented [11, 12]. Upon species identification, female sand flies of the same species and collected at the same time point from the same trap were homogenised in groups of five and kept frozen at − 20 °C until further molecular analysis.

### Haematology

A complete blood count (CBC) was performed as a broad screening test in order to support the assessment of each dog’s health status. This screening included: haematocrit, haemoglobin, mean corpuscular volume, mean corpuscular haemoglobin, mean corpuscular haemoglobin concentration, platelet count, red cell distribution width, red blood cell count and white blood cell count. The CBCs were performed by the Diagnostic Laboratory, Department of Clinical Studies, School of Veterinary Medicine, Aristotle University of Thessaloniki, Greece.

### Serology

An enzyme-linked indirect immunosorbent assay (ELISA) was performed on sera samples for the detection of specific antibodies of *L. infantum* (INGEZIM® Leishmania; Ingenasa, Madrid, Spain), *Ehrlichia canis* (INGEZIM® *Ehrlichia*; Ingenasa) and *Anaplasma phagocytophilum* (*Anaplasma*-ELISA Dog; AFOSA GmbH, Blankenfelde-Mahlow, Germany). All assays were performed following the respective manufacturer’s instructions. All sera samples collected on all SDs were tested for all of the above pathogens.

### Molecular analyses

Genomic DNA was extracted from the EDTA-blood samples, conjunctival swabs and sand flies using the DNeasy Blood & Tissue Kit (Qiagen GmbH, Hilden, Germany), according to the manufacturer’s instructions.

Blood samples, conjunctival swabs and sand fly DNA samples were tested for *L. infantum* DNA using a TaqMan real-time quantitative PCR (qPCR) assay that targeted a 120-bp fragment of the kinetoplast minicircle DNA, as previously reported [13]. A positive (reference DNA sample from blood, conjunctival swab and sand fly DNA, respectively) and a negative (PCR grade water) control were included in each qPCR run.

The qPCR was performed only on samples collected on SDs 0 and 630 and, in addition, on DNA blood samples at all time points from: (i) all dogs that tested positive at the beginning of the study and became negative during the study; (ii) all dogs that tested positive by ELISA only; and (iii) all dogs that tested only as low positive on qPCRs performed on swab DNA samples on SD 630.

For *E. canis, Anaplasma platys* and *A. phagocytophilum*, specific PCR tests were performed on all DNA from blood samples on SD 0 and SD 630 collected from dogs with at least 1 year follow-up. To determine the presence of *E. canis,* a PCR targeting a 345-bp fragment of the 16S ribosomal RNA (rRNA) gene [14] of various species, including *E. canis, Ehrlichia chaffeensis, Ehrlichia muris, Ehrlichia ruminantium, A. phagocytophilum, A. platys, Anaplasma marginale, Anaplasma centrale, Wolbachia pipentis, Neorickettsia sennetsu, Neorickettsia risticii* and *Neorickettsia helminthoeca*, was performed according to previously described thermal-cycling conditions [15].

To identify the presence of *Anaplasma* DNA, we performed species-specific nested PCRs for *A. platys* and *A. phagocytophilum* DNA targeting a 678-bp and a 546-bp fragment of the 16S rRNA gene, respectively, according to previously described protocols [16].

A positive (*E. canis* and *A. platys* or *A. phagocytophilum* reference DNA sample) and a negative (PCR grade water) control were included in each PCR run. Amplification products were visualised on 1.5% agarose gels stained with ethidium bromide.

PCR products (from samples positive for *E. canis* and *A. platys/phagocytophilum*) were sent to a commercial service (CeMIA SA, Larissa, Greece) for purification and sequencing on both strands (Sanger sequencing). The results were assembled with Seqman 8.1 software (DNASTAR, Madison, WI, USA). Assembled sequences were aligned using the Basic Local Alignment Tool (BLAST) and compared with reference sequences using the MegAlign application of the Lasergene software package (DNASTAR).

### Efficacy criteria

The efficacy of the Seresto® (10% w/w imidacloprid/4.5% w/w flumethrin) collar was assessed based on the results of the various serological and molecular tests to detect exposure to CVBD pathogens (*L. infantum, Ehrlichia* spp.*, Anaplasma* spp.) and ectoparasite counts (fleas and ticks) performed throughout the duration of the study.

A dog was considered positive for a given pathogen if exposure to the latter had been confirmed, either directly or indirectly, in one or more of the diagnostic tests. Dogs that tested negative for a particular or all CVBPs at study start were considered to be protected if the latter status was maintained until the final visit. The product was considered to be effective against fleas and ticks when the infestation intensity remained ≤ 1.

Only dogs that were followed up for at least 1 year (i.e. inclusion on SD 0 or SD 210 and at least 2 more time points of follow-up after inclusion) were included in the analyses. Dogs were excluded from the analyses if they tested positive in any of the CVBP tests performed at the day of inclusion. Dogs that tested positive in any of the tests at inclusion or/and during the study remained collared and were followed up like all other dogs.

Dogs were considered to be a treatment success if they had either no positive result in any of the follow-up tests or had only one positive ELISA result in any of the follow-up tests (the 1 positive result was considered to be a random positive test, possibly due to cross-reaction). Dogs were considered to be a treatment failure if they had either ≥ 2 positive ELISA results at follow-up time points, or a positive PCR result at the last evaluation time point.

### Statistical analyses

The statistical unit was the individual dog.

#### Descriptive analyses

Descriptive statistical analyses were performed on data from the clinical examinations, daily health assessments, BW and hair length measures, safety observations (i.e. administration observations, local tolerance observations and adverse effects), infestation intensity for fleas and ticks, and sand fly counts and serological and molecular analyses. Median (Mdn), mean (M) and standard deviation (SD) values were calculated for genome copy values of *L. infantum* obtained by qPCR.

#### Percentage of population protected from transmission of CVBPs

The percentage of the study population protected from the transmission of CVBPs was calculated for each of the tested pathogens using the following equation:

$${\text{Percentage of population protected (\% ) }} = \frac{{{\text{Mpre }} - {\text{ Mfail }} \times { 100}}}{{{\text{Mpre}}}}$$

where Mpre = the number of dogs that were negative on the day of inclusion into the study (SD 0 or SD 210); and Mfail = the number of dogs that were considered to be a treatment failure in the overall evaluation after completion of the study.

#### Incidence density rate

The incidence density rate (IDR) was calculated as per the equation below, taking into account the total amount of time each dog spent in the study.

$${{\text{IDR per year}} = \frac{{\text{Number of treatment failures in overall evaluation}}}{{{\text{Time each dog was observed }}\left( {\text{in years}} \right){\text{, totalled for all dogs}}}} \times 100\,} $$

## Results

### Study population

A total of 479 privately owned dogs were enrolled in the study (206 in Heraklion and 273 in Leros). Most of the included dogs were mongrels (*n* = 289), followed by dogs identified as Pointer (*n* = 36), Setter (*n* = 23), Greek Harehound (Geka; *n* = 21), Cretan Hound (*n* = 14), Beagle (*n* = 12), Kurzhaar (*n* = 10) and several other breeds. Of these 479 dogs, 102 were living mostly indoors; the majority (*n* = 377) were living outdoors in the backyard of the owner or in kennels. Age at inclusion ranged from 3 months to 15 years, and 268 dogs were females (211 males). There were 262 dogs with short hair (< 2 cm), 94 with a medium length hair (2–5 cm) and 122 with medium to long or long hair (> 5 cm). The BW ranged from 2.4 to 69.0 kg at inclusion.

Overall, 90 dogs were removed from the study at different time points. None of the removals were issues related to collar administration. Specifically, two dogs lost the collar and there was a failure in replacing it in < 48 h; 19 dogs died during the study duration due to various causes, such as severe chronic diseases or injuries (e.g. car accidents); 59 were removed based on their owner decision or due to non-complian; and 10 other dogs were removed for various other reasons. The Seresto® collars were safe and well tolerated by all participating dogs. Overall, there were six mild and one moderate adverse events reported during the study (Additional file 1: Table S1). All adverse events except one were considered unlikely to be related to treatment administration, the one exception concerned a dog which presented with hair loss and erythema on the ventral neck below the collar on SD 210.

### Clinical examination

At inclusion several dogs (*n* = 50) showed clinical signs potentially related to CVBDs, such as skin lesions, weight loss, enlarged lymph nodes, pale mucous membranes, conjunctivitis and onychogryphosis. Some clinical signs were also observed in several dogs (*n* = 136) at one or more of the time points during the scheduled observations/examinations after SD 0, of which some were likely to have been associated to CVBDs. None of the enrolled dogs showed any severe clinical signs related to CVBDs to an extent to necessitate treatment or removal from the study.

All CBC results were either within the laboratory reference range values or in line with biological variations that did not correlate with clinical findings or raise concern. Increasing BW in individual dogs was subject to normal variation throughout the observation period.

### Ectoparasite infestations

Most dogs with fleas and ticks at inclusion were from Leros, with some having severe infestations. In total, 72 dogs had at least one tick and 37 had at least one flea. After placement of the collar, only one dog had a low number (1–3) of ticks on SD 420 and another had one dead tick on SD 630. The vast majority of ticks collected from dogs of both sites were identified as *Rhipicephalus sanguineus* sensu lato (*R. sanguineus* s.l.), with only two *R. turanicus* sensu lato (*R. turanicus* s.l) ticks collected from dogs living in Leros. After placement of the collar, six dogs were found with a low number (1–5) of fleas (3 dogs each on SD 210 and SD 420) and one dog had a high number (> 20) of fleas on SD 420). All fleas were identified as *Ctenocephalides felis felis.*

### Sand fly collection

A total of 414 sand flies (163 female, 251 male) belonging to nine species were captured during the trapping events. The predominant species of sand flies, based on collected female sand flies, were *Phlebotomus neglectus* (49 specimens) and *Sergentomyia minuta* (30 specimens), followed by *Phlebotomus tobbi* (24 specimens); *Phlebotomus perfiliewi, Phlebotomus similis*, *Phlebotomus simici*, *Phlebotomus mascittii*, *Phlebotomus papatasi* and *Sergentomyia dentata* were also identified. None of the sand flies was found to harbour *L. infantum* DNA based on the results of the molecular analyses.

### Serology

At inclusion in the study, no antibodies against any of the pathogens tested were detected in the majority of dogs (79.1%). At the precise time of inclusion, 93.5% of the dogs (191 from Crete, 257 from Leros) tested negative for the presence of *L. infantum* antibodies, 82.2% (164 dogs from Crete, 230 dogs from Leros) did not have any antibodies for *E. canis* and 98.1% (203 dogs from Crete, 267 dogs from Leros) tested negative for *A. phagocytophilum* antibodies.

Of the dogs that completed the study, 96.7% (154 dogs from Crete, 230 dogs from Leros) tested negative for *L. infantum* antibodies, 93% (133 dogs from Crete, 200 dogs from Leros) did not harbour any antibodies for *E. canis* and 87.2% (155 dogs from Crete, 205 dogs from Leros) tested negative for the presence of *A. phagocytophilum* antibodies.

### Molecular analyses

At inclusion (SD 0) almost all dogs (94.1%) tested negative for the presence of the *L. infantum* genome in either the blood or swab samples. Of the dogs that tested positive for the *L. infantum* genome in the PCR assay, the majority tested positive in the swab samples only (57.1%), followed by dogs that tested positive in both the swab and blood samples (32.1%); only a small number of samples tested positive by the PCR assay in blood samples only (10.7%). For the dog population from Crete, blood samples found to be positive by qPCR had a very low quantity of the parasite genome, ranging between 1.024 and 94 copies/ml blood (Mdn: 35.7, M: 41.61, SD: 43.37); in swab samples, the quantity of the parasite genome ranged from 0.065 to 6850 copies/ml phosphate-buffered saline (PBS; Mdn: 41.5, M: 1036.79, SD: 2206.53). For the dog population from Leros, for blood samples that tested positive in the qPCR assay, the quantity of *L. infantum* genome ranged between 2.6 and 3086 copies/ml blood (Mdn: 264, M: 765.23, SD: 1139.60), while in the swab samples, the quantity was higher, ranging from 0.2 to 14,980 genome copies/ml PBS (Mdn: 3.00, M: 1917.82, SD: 4466.94).

Of the dogs that completed the study most remained negative (90.7%) according to the qPCR assays of either the blood or swab samples. Again, the majority of dogs tested positive for the swab samples only (80.6%), followed by dogs that tested positive for the swab and blood samples (13.9%); agains, only a small number of samples tested positive by the PCR assay in blood samples only (5.6%).

The quantity of *L. infantum* genome in positive blood samples from dogs living in Crete ranged between 28 and 4864 copies/ml blood (Mdn: 1436, M: 1815.14, SD: 1583.68), while the quantity ranged between 2 and 4597.5 copies/ml PBS (Mdn: 66, M: 532.05, SD: 1356.80) in positive swab samples. For the dog population from Leros, the quantity of *L. infantum* in positive blood samples ranged between 2 and 398 copies/ml blood (Mdn: 30, M: 43.33, SD: 49.37), while for positive swab samples, the quantity ranged between 1 and 391,500 copies/ml PBS (Mdn: 40, M: 15,064.15, SD: 71,400.73).

At inclusion, all dogs from Crete were positive for the presence of *E. canis* according to the PCR results, while three dogs from Leros were positive. Of the dogs that completed the study, again all dogs from Crete were negative at inclusion according to the PCR assay; in Leros, *E. canis* DNA was detected in two dogs at the end of the study.

At inclusion, *Anaplasma* spp. DNA was not detected in any of the dogs included from Crete, however *Anaplasma* spp. DNA was detected in 14 of the dogs included from Leros. Of the dogs that completed the study, only one dog in Crete was positive for *A. platys* DNA, while *A. platys* DNA was detected in 22 dogs in Leros.

Representative sequences of both *E. canis* and *A. platys* were deposited in the GenBank under the accession numbers MN922608-MN962611.

### Percentage of population protected from *L. infantum*

The population for the evaluation of *L. infantum* transmission consisted of 397 dogs (160 in Crete and 237 in Leros). In total, 39 new infections with *L. infantum* occurred throughout the observation period (10 in Crete, 29 in Leros). This corresponds to an overall efficacy in the prevention of transmission (protection) of 90.2%. The efficacy was statistically significantly different (Chi-square test, *χ*^2^ = 3.8639, *df* = 1, *P* = 0.0493) between the two populations in Crete (93.7%) and Leros (87.8%) (Table 1).

**Table 1:** Percentage of dog study population protected from *Leishmania infantum* transmission for the duration of the entire study

Study site	Study period (*n* days)	Number of dogs testing positive for *L. infantum*	Total number of dogs included in study	Percentage of dogs testing positive for *L. infantum*	Percentage of protection
Crete	0–630	10	160	6.25	93.75
Leros	0–630	29	237	12.24	87.76
Combined	0–630	39	397	9.82	90.18

### Specific follow-up for *L. infantum*

A noteworthy observation was made for a subset of dogs from a single household in Leros. Twenty-six dogs were living in this household, of which four tested positive and 22 tested negative for *Leishmania* infection at inclusion. Of the 22 which tested negative at inclusion, 16 tested negative for *Leishmania* throughout the study, with the exception that all 16 had a positive result for the qPCR on conjunctival swab samples on SD 630, which showed a low quantity of parasite genome, ranging between 1 and 333.5 copies/ml PBS (Mdn: 9.5, M: 46, SD: 90.17).

For the dogs in this specific household, we considered it justified to prolong the study duration with the objective to monitor whether these dogs would ultimately establish a *Leishmania* infection. After SD 630, these dogs were followed up for an additional 7 months; on SD 630 the collars were replaced for a further 7-month period, and all previously described parasitological and clinical procedures were followed as described above. Test results at the end of follow-up period showed that all 16 dogs which tested positive on the swab qPCR on SD 630 were negative and remained negative for all of the other tests performed at this time.

Due to the above, it was decided to perform an additional analysis for the evaluation of efficacy in the prevention of transmission (protection) for *Leishmania* excluding this subset of dogs, resulting in 215 dogs for the evaluation in Leros, of which 13 were newly infected with *Leishmania* during the study period. Based on this population subset, efficacy for the risk reduction of transmission for *Leishmania* in Leros was 93.9% and overall protection 93.9%. In this scenario, the efficacy was statistically not significantly different (Chi-square test, *χ*^2^ = 0.0066, *df* = 1, *P* = 0.9353) between the two populations in Crete and Leros (Table 2).

**Table 2:** Percentage of dog study population protected from *Leishmania infantum* transmission during the whole study duration when dogs of a specific household in Leros (*n* = 22) were excluded from the analysis population

Study site	Study period (days)	Number of dogs testing positive for *L. infantumn*	Total number of dogs	Percentage of dogs testing positive for *L. infantum*	Percentage of protection
Crete	0–630	10	160	6.25	93.75
Leros	0–630	13	215	6.05	93.95
Combined	0–630	23	375	6.13	93.87

*N* Total number of dogs, *pos* positive

The overall IDR was 5.9% (3.8% in Crete, 7.4% in Leros) (Table 3). When the dogs of the one specific household were excluded from analysis, the IDR decreased to 3.7% in Leros, resulting in an overall IDR of 3.7% (Table 4).

**Table 3:** Incidence density rate per year for all pathogens in each study site and combined

Study site	Pathogen	Number of dogs testing positive for pathogen	Total number of dogs	Duration within study			IDR
				365 days	420 days	630 days	
Crete	*Anaplasma *spp.	19	174	3	22	149	6.66
	*Ehrlichia canis*	8	141	2	20	119	3.47
	*Leishmania infantum*	10	160	2	18	140	3.78
Leros	*Anaplasma *spp.	34	239	14	24	201	8.75
	*Ehrlichia canis*	17	216	11	20	185	4.81
	*Leishmania infantum*	29	237	11	18	208	7.42
Combined	*Anaplasma *spp.	53	413	17	46	350	7.86
	*Ehrlichia canis*	25	357	13	40	304	4.28
	*Leishmania infantum*	39	397	13	36	348	5.95

*IDR* Incidence density rate per year for 100 dogs

**Table 4:** Incidence density rate per year for *Leishmania infantum* in each study site and combined, when dogs of a specific household (*n* = 22) in Leros were excluded from the analysis

Study site	Pathogen	Number of dogs testing positive for pathogen	Total number of dogs	Duration within study			IDR
				365 days	420 days	630 days	
Crete	*L. infantum*	10	160	2	18	140	3.78
Leros		13	215	11	18	186	3.69
Combined		23	375	13	36	326	3.73

### Percentage of population protected from tick-borne diseases (*Anaplasma* spp. and *E. canis*)

A total of 413 dogs (174 in Crete, 239 in Leros) were included in the evaluation of *Anaplasma *spp. transmission. In total, 53 new infections with *Anaplasma *spp. occurred throughout the observation period (19 in Crete, 34 in Leros), corresponding to an overall efficacy in the prevention of transmission (protection) of 87.2%. The efficacy was not statistically significantly different (Chi-square test, *χ*^2^ = 0.9841, *df* = 1, *P* = 0.3212) between the two populations in Crete (89.1%) and Leros (85.8%) (Table 5).

**Table 5:** Percentage of dog study population protected from *Anaplasma *spp. transmission during the entire study

Study site	Study period (days)	Number of dogs testing positive for *Anaplasma *spp.	Total number of dogs	Percentage of dogs testing positive for *Anaplasma *spp.	Percentage of protection
Crete	0–630	19	174	10.92	89.08
Leros	0–630	34	239	14.23	85.77
Combined	0–630	53	413	12.83	87.17

*N* Total number of dogs, *pos* positive

The population for the evaluation of *E. canis* transmission consisted of 357 dogs (141 in Crete, 216 in Leros). In total, 25 new infections with *E. canis* occurred throughout the observation period (8 in Crete, 17 in Leros), corresponding to an overall efficacy in the prevention of transmission (protection) of 93%. The efficacy was not statistically significantly different (Chi-square test, *χ*^2^ = 0.6321, *df* = 1, *P* = 0.4266) between the two populations in Crete (94.3%) and Leros (92.1%) (Table 6).

**Table 6:** Percentage of dog study population protected from *Ehrlichia canis* transmission during the entire study

Study site	Stduy period (days)	Number of dogs testing positive for *E. canis*	Total number of dogs	Percentage of dogs testing positive for *E. canis*	Percentage of protection
Crete	0–630	8	141	5.67	94.33
Leros	0–630	17	216	7.87	92.13
Combined	0–630	25	357	7.00	93.00

*N* Total number of dogs, *pos* positive

The overall IDR was 7.9% (6.7% in Crete and 8.7% in Leros) for *Anaplasma* spp. and 4.3% (3.5% in Crete and 4.8% in Leros) for *E. canis* (Table 3).

## Discussion

This study was designed to evaluate the protective capacity of the Seresto® collar on the transmission of vector-borne pathogens over a period of almost 2 years continuous collar use in two highly endemic areas under field conditions. To our knowledge this is the only field study to date that has enrolled such a large number of dogs (479 privately owned dogs) and for such a long period (study duration: at least 21 months).

Specifically, for *L. infantum*, the prevention of transmission was calculated as 90.2% combining cases in both locations (Crete and Leros), with a divergence between the two sites (93.7% in Crete, 87.8% in Leros). Close examination of these data revealed that the lower protection rate recorded in Leros was due to a subset of 16 dogs from a single household whose swab samples tested positive with qPCR only on SD 630, with a low parasitic burden, with all other previous analyses being negative as well as analyses carried out at a later follow-up. To further investigate if this result was suggestive of an active infection or not, all dogs of the specific household were followed up for an extra period of 7 months. Since these 16 dogs tested negative in all analyses other than the test on the conjunctival swab in the follow-up period, we ran a second statistical analysis excluding all dogs of this household. Reverse transcription-qPCR (qRT-PCR) on conjunctival swabs has proven to be a very sensitive non-invasive technique to screen dogs for leishmaniosis, as it has the potential to detect infection earlier than if it were performed on other tissues, even before any symptoms become evident [21–23]. However, it has been suggested that a positive result only by qRT-PCR on conjunctival swabs, in the absence of a positive result with any other diagnostic test or the presence of any clinical sign, should be considered with caution [24]. With the exclusion of this subsect, *L. infantum* efficacy for Leros increased from 87.8% to 93.9%, which is in total agreement with the findings from Crete.

The findings in the specific household in Leros represent a scientifically interesting phenomenon. There were 26 dogs living together in this household, all of which were enrolled in the study; of these, four tested positive at study inclusion. The study protocol was the same for these four positive dogs as for all other dogs enrolled in the study, but only one of these dogs continued to be positive throughout the study. This dog had a high parasitic burden (as expressed in number of copies in the qPCR and ELISA antibody levels), however it never developed symptoms to justify treatment. This dog, which lived in close proximity the 25 other dogs of the household, is assumed having raised infection pressure within this community, acting as an infection “super spreader” [25]. This scenario is supported by the fact that insects identify potential host animals by combinations of visual, thermal, tactile and chemical (host odour) cues produced by the host, and are therefore found close to dog communities (this is more obvious for sand flies who have not a great ability to fly long distances) [26]. Moreover, it has been recently documented that the odour of *L. infantum*-infected dogs is more attractive to female sand flies than the odour of uninfected dogs, leading to enhanced infection and transmission opportunities for the parasite [27]. Our results suggest that the above-mentioned 16 dogs had some contact with *Leishmania* parasites due to the high infection pressure of the environment, but that the infection pressure was not sufficient high for the infection to be established.

The entomological survey confirmed the presence of competent vectors of *L. infantum* in all the monitored locations, namely *P. neglectus, P. tobbi* and *P. perfiliewi*, all of which are known to be important vectors of *L. infantum* in the Mediterranean basin [28] and to be previously recorded in Greece [29–32]. The results from the survey confirm that the environment in both areas is favourable for completion of the *L. infantum* life-cycle. While we did not isolate the parasite in the sand flies themselves, this can probably be explained by an inadequate number of females being captured and examined during the study. Similar studies in the eastern Mediterranean region have reported sand fly prevalences of 7.9 to 14.5%.

As expected based on previously published data, the vast majority of ticks collected belonged to *R. sanguineus* s.l. species and the fleas identified were *C. felis felis* [33–36]. Protection against ticks and fleas was demonstrated to be very high, with only two dogs having a low number of ticks and seven dogs having mostly low numbers of fleas at one evaluation time point. These findings agree with those from previous field studies [19, 20, 37]. These results were particularly evident in all dogs living in rural settlements and in open door spaces, such as hunting dogs of Leros island. During selection of the study location [3], dogs showed massive infestations of ticks and fleas due to poor owner management, primarily due to economic and cultural reasons. The efficacy of the Seresto® collar was also maintained when it was smeared with mud and in dogs which had frequent contact with water puddles due to hunting activity. Owners’ feedback on the easy application, usefulness and tolerability of the collar was highly positive. Regarding the tick-borne pathogens investigated in the current study, the overall (combining results from Crete and Leros) protective effect was calculated as 87.2% and 93% for *Anaplasma* spp*.* and* E. canis*, respectively. The efficacy of the Seresto® collar against *R. sanguineus* s.l. ticks can also reduce the risk for infection with pathogens transmitted by those ticks, such as *E. canis* and *A. platys,* as previously demonstrated under controlled infection and field study conditions [38, 39]. Therefore, our results are consistent with previous findings, suggesting that the Seresto® collar can protect dogs from ticks and fleas and reduce the risk of pathogen transmission [40]. This risk reduction, as seen in our long-term field study with privately owned dogs, is remarkable as in such a setting factors such as owner compliance (possible removal or not immediate replacement of the collar) could negatively influence the final results.

Overall, the results of our study involving a large population of dogs confirm that long-term protection against ticks, fleas and the most common CVBPs can be achieved in an endemic area by placing the Seresto® collar around the necks of the dogs. Such long-term CVBP prevention not only provides the dogs with health/welfare protection, but can also help to reduce potential risk for human health. For continuous prevention in such an environment it is highly important that the collar be worn continuously and that the dog keeps wearing the collar since the risk of infection by CVBPs upon removal of the collars increases quickly [39, 41].

## Conclusions

Treatment with Seresto® collars proved to be highly efficacious and safe, confirming the results of previous field studies in which protection against transmission of various CVBPs was studied in different environment and animals [17–20].

## Supplementary Information

**Additional file 1:** **Table S1.** Records of adverse events and their outcome during the whole study duration.

## Data Availability

The dataset supporting the conclusions of this article is included within the article. Due to commercial confidentiality of the research, data not included in the manuscript can only be made available to researchers, subject to a non-disclosure agreement.

## Abbreviations

BW: Body weight
CBC: Complete blood count
CVBPs: Canine vector-borne pathogens
ELISA: Enzyme-linked immunosorbent assay
IDR: Incidence density rate
M: Mean
Mdn: Median
qPCR: Quantitative PCR
SD: Study day

## References

[1] Otranto D, Wall R (2008). New strategies for the control of arthropod vectors of disease in dogs and cats. Med Vet Entomol.

[2] Baneth G, Bourdeau P, Bourdoiseau G, Bowman D, Breitschwerdt E, Capelli G (2012). Vector-borne diseases—Constant challenge for practicing veterinarians: recommendations from the CVBD World Forum. Parasit Vectors.

[3] Kostopoulou D, Gizzarelli M, Ligda P, Foglia Manzillo V, Saratsi K, Montagnaro S (2020). Mapping the canine vector-borne disease risk in a Mediterranean area. Parasit Vectors.

[4] Wright I, Jongejan F, Marcondes M, Peregrine A, Baneth G, Bourdeau P (2020). Parasites and vector-borne diseases disseminated by rehomed dogs. Parasit Vectors.

[5] Schäfer I, Volkmann M, Beelitz P, Merle R, Müller E, Kohn B (2019). Retrospective analysis of vector-borne infections in dogs after travelling to endemic areas (2007–2018). Vet Parasitol.

[6] Maia C, Cardoso L (2015). Spread of *Leishmania infantum* in Europe with dog travelling. Vet Parasitol.

[7] Tamponi C, Scarpa F, Carta S, Knoll S, Sanna D, Gai C (2021). Seroprevalence and risk factors associated with *Leishmania infantum* in dogs in Sardinia (Italy), an endemic island for leishmaniasis. Parasitol Res.

[8] Müller A, Montoya A, Escacena C, de la Cruz M, Junco A, Iriso A (2022). *Leishmania infantum* infection serosurveillance in stray dogs inhabiting the Madrid community: 2007–2018. Parasit Vectors.

[9] Estrada-Pena A, Bouattour A, Camicas J, Walker AR (2004). Ticks of domestic animals in the mediterranean region: a guide to identification of species.

[10] Walker J, Keirans J, Horak I (2000). Genus *Rhipicephalus* (Acari, Ixodidae). A guide to the brown ticks of the world.

[11] Theodor O. Psychodidae-phlebotominae. In: Lindner E, editor. Die Flieg Der Palaerktischen Reg. Lieferung 201. Stuttgart:E. Schweizerbart’sche Verlagsbuchhandlung; 1958. p. 1–55.

[12] Killick-Kendrick R, Tang Y, Killick-Kendrick M, Sang D, Sirdar M, Ke L (1991). The identification of female sandflies of the subgenus *Larroussius* by the morphology of the spermathecal ducts. Parasitologia.

[13] Francino O, Altet L, Sánchez-Robert E, Rodriguez A, Solano-Gallego L, Alberola J (2006). Advantages of real-time PCR assay for diagnosis and monitoring of canine leishmaniosis. Vet Parasitol.

[14] Inokuma H, Raoult D, Brouqui P (2000). Detection of *Ehrlichia platys* DNA in brown dog ticks (*Rhipicephalus sanguineus*) in Okinawa Island. Japan J Clin Microbiol.

[15] Brown GK, Martin AR, Roberts TK, Aitken RJ (2001). Detection of *Ehrlichia platys* in dogs in Australia. Aust Vet J.

[16] Springer A, Montenegro VM, Schicht S, Globokar Vrohvec M, Pantchev N, Balzer J (2019). Seroprevalence and current infections of canine vector-borne diseases in Costa Rica. Front Vet Sci.

[17] Brianti E, Napoli E, Gaglio G, Falsone L, Giannetto S, Solari Basano F (2016). Field evaluation of two different treatment approaches and their ability to control fleas and prevent canine leishmaniosis in a highly endemic area. PLoS Negl Trop Dis.

[18] Brianti E, Gaglio G, Napoli E, Falsone L, Prudente C, Solari Basano F (2014). Efficacy of a slow-release imidacloprid (10%)/flumethrin (4.5%) collar for the prevention of canine leishmaniosis. Parasit Vectors.

[19] Brianti E, Falsone L, Napoli E, Prudente C, Gaglio G, Giannetto S (2013). Efficacy of a combination of 10% imidacloprid and 4.5% flumethrin (Seresto®) in slow release collars to control ticks and fleas in highly infested dog communities. Parasit Vectors.

[20] Stanneck D, Rass J, Radeloff I, Kruedewagen E, Le Sueur C, Hellmann K (2012). Evaluation of the long-term efficacy and safety of an imidacloprid 10%/flumethrin 4.5% polymer matrix collar (Seresto®) in dogs and cats naturally infested with fleas and/or ticks in multicentre clinical field studies in Europe. Parasit Vectors.

[21] Leite RS, de Ferreira SA, Ituassu LT, de Melo MN, de Andrade ASR (2010). PCR diagnosis of visceral leishmaniasis in asymptomatic dogs using conjunctival swab samples. Vet Parasitol.

[22] Strauss-Ayali D, Jaffe CL, Burshtain O, Gonen L, Baneth G (2004). Polymerase chain reaction using noninvasively obtained samples, for the detection of *Leishmania infantum* DNA in dogs. J Infect Dis.

[23] Gondim C, Ferreira S, Vasconcelos B, Wouters F, Fujiwara R, de Castro J (2022). Visceral leishmaniasis in a recent transmission region: 27.4% infectivity rate among seronegative dogs. Parasitology.

[24] Cavalera MA, Zatelli A, Donghia R, Mendoza-Roldan JA, Gernone F, Otranto D (2022). Conjunctival swab real time-PCR in *Leishmania infantum* seropositive dogs: diagnostic and prognostic values. Biology.

[25] Duthie MS, Lison A, Courtenay O (2018). Advances toward diagnostic tools for managing zoonotic visceral leishmaniasis. Trends Parasitol.

[26] Lehane M (2005). The biology of blood-sucking in insects.

[27] Staniek ME, Hamilton JGC (2021). Odour of domestic dogs infected with *Leishmania infantum* is attractive to female but not male sand flies: evidence for parasite manipulation. PLoS Pathog.

[28] Cecílio P, Cordeiro-da-Silva A, Oliveira F (2022). Sand flies: basic information on the vectors of leishmaniasis and their interactions with *Leishmania* parasites. Commun Biol.

[29] Alten B, Maia C, Afonso MO, Campino L, Jiménez M, González E (2016). Seasonal dynamics of Phlebotomine sand fly species proven vectors of Mediterranean leishmaniasis Caused by *Leishmania infantum*. PLoS Negl Trop Dis.

[30] Dvořák V, Tsirigotakis N, Pavlou C, Dokianakis E, Akhoundi M, Halada P (2020). Sand fly fauna of Crete and the description of *Phlebotomus* (*Adlerius*) *creticus* n. sp. (Diptera: Psychodidae). Parasit Vectors.

[31] Giantsis IA, Beleri S, Balatsos G, Karras V, Patsoula E, Papachristos D (2021). Sand fly (Diptera: Psychodidae: Phlebotominae) population dynamics and natural *Leishmania* infections in Attica region, Greece. J Med Entomol.

[32] Fotakis EA, Giantsis IA, Castells Sierra J, Tanti F, Balaska S, Mavridis K (2020). Population dynamics, pathogen detection and insecticide resistance of mosquito and sand fly in refugee camps. Greece Infect Dis Poverty.

[33] Diakou A, Di Cesare A, Morelli S, Colombo M, Halos L, Simonato G (2019). Endoparasites and vector-borne pathogens in dogs from Greek islands: pathogen distribution and zoonotic implications. PLoS Negl Trop Dis.

[34] Efstratiou A, Karanis G, Karanis P (2021). Tick-borne pathogens and diseases in Greece. Microorganisms.

[35] Angelou A, Gelasakis AI, Verde N, Pantchev N, Schaper R, Chandrashekar R (2019). Prevalence and risk factors for selected canine vector-borne diseases in Greece. Parasit Vectors.

[36] Latrofa MS, Angelou A, Giannelli A, Annoscia G, Ravagnan S, Dantas-Torres F (2017). Ticks and associated pathogens in dogs from Greece. Parasit Vectors.

[37] Horak IG, Fourie JJ, Stanneck D (2012). Efficacy of slow-release collar formulations of imidacloprid/flumethrin and deltamethrin and of spot-on formulations of fipronil/(s)-Methoprene, dinotefuran/pyriproxyfen/permethrin and (s)-methoprene/amitraz/fipronil against *Rhipicephalus sanguineus* and *Ctenocephalides felis felis* on dogs. Parasit Vectors.

[38] Stanneck D, Fourie JJ (2013). Imidacloprid 10%/ flumethrin 4.5% collars (Seresto®, Bayer) successfully prevent long-term transmission of *Ehrlichia canis* by infected *Rhipicephalus sanguineus* ticks to dogs. Parasitol Res.

[39] Dantas-Torres F, Capelli G, Giannelli A, Ramos RAN, Lia RP, Cantacessi C (2013). Efficacy of an imidacloprid/flumethrin collar against fleas, ticks and tick-borne pathogens in dogs. Parasit Vectors.

[40] Dantas-Torres F (2010). Biology and ecology of the brown dog tick *Rhipicephalus sanguineus*. Parasit Vectors.

[41] Otranto D, de Caprariis D, Lia RP, Tarallo V, Lorusso V, Testini G (2010). Prevention of endemic canine vector-borne diseases using imidacloprid 10% and permethrin 50% in young dogs: a longitudinal field study. Vet Parasitol.

