# Supplementary material for: Determination of the effect of collars containing 10% w/w imidacloprid and 4.5% w/w flumethrin (Seresto®) on the incidence of Leishmania and other canine vector-borne pathogen infections in Greece
Source: Parasit Vectors. 2023 Mar 7;16:89. doi: 10.1186/s13071-023-05678-4 (PMC9990277; doi:10.1186/s13071-023-05678-4)
Supplement: Supplementary file 1 — Additional file 1: Table S1. Records of adverse events and their outcome during the whole study duration. [file 13071_2023_5678_MOESM1_ESM.docx]

**Additional file 1: Table S1: Records of adverse events and their outcome during the whole study duration**

| ID | Causal  Relationship | Severity | Time post treatment | Adverse Event | Outcome | VEDDRA^1^ code | ABON^2^ |
| --- | --- | --- | --- | --- | --- | --- | --- |
| 1 | Probable | Mild | 210 days | hair loss and erythema on ventral neck below collar | No information received | Application site reactions - Application site erythema | B |
| 2 | Unlikely | Mild | 5 hours | skin rash due to clipping | Recovery | Epidermal and dermal disorders - Dermatitis and eczema | O |
| 3 |  |  | 6 days | external otitis in the right ear; pain, ear scratching | Recovery within 7 days | External ear disorders - Otitis externa |  |
| 4 |  |  | 9 days | Gastroenteritis; vomiting, diarrhea, loss of appetite | Recovery within 2 days | Other digestive tract disorders - Gastroenteritis |  |
| 5 |  |  | 206 days | Wound during hunting | Recovery | General signs or symptoms - Trauma NOS |  |
| 6 |  |  | 207 days | blood at the urine | No information received | Urinary tract disorders- Urinary tract bleeding |  |
| 7 |  | Moderate | 206 days | Otitis; chronic otitis on the left ear | No information received | External ear disorders - Otitis externa |  |
| 8 |  | Serious | 8 days | Death; dog was vomiting the last week and also displayed melena. | Death | Death - Death |  |
| 9 |  |  | 15 days | Death; car accident |  | Death - Death |  |
| 10 |  |  | 46 days | Collapse / paralysis; the animal collapsed. When examined displayed weakness and loss of appetite. |  | General signs or symptoms - Collapse NOS |  |
| 11 |  |  | 72 days | Death; breast tumor |  | Mammary gland neoplasms - Mammary gland neoplasm NOS |  |
| 12 |  |  | 138 days | Death; animal was found positive to *Leishmania* tests performed since the beginning of the trial (before placing the collar). |  | Death - Death |  |
| 13 |  |  | 156 days | Death; dog was brought to the clinic with anorexia, weakness, lethargy, depression and vomiting. It died 20 minutes later. |  | Death - Death |  |
| 14 |  |  | 181 days | Death; acute gastroenteritis |  | Other digestive tract disorders - Gastroenteritis |  |
| 15 |  |  | 202 days | Death; heart and renal failure |  | Multi-organ failure - Multi-organ failure NOS |  |
| 16 |  |  | 206 days | Death; car accident |  | Death - Death |  |
| 17 |  |  | 209 days | Death; dog was found dead, after many weeks of coughing. |  | Death - Death |  |
| 18 |  |  | 212 days | Death; septic shock from injures and bites left untreated. |  | General signs or symptoms - Septicaemia |  |
| 19 |  |  | 218 days | Death; the dog was vomiting for 2 hours, suffering from muscle spasms and died. |  | Death - Death |  |
| 20 |  |  | 227 days | Death; car accident |  | Death - Death |  |
| 21 |  |  | 29 days | Death; dog was found dead by the owner. |  | Death - Death | N |
| 22 |  |  | 122 days | Death |  |  |  |
| 23 |  |  | 151 days | Death; dog was found dead by the owners without any previous clinical symptoms. |  |  |  |
| 24 |  |  | 196 days | Death; dog was found dead by the owner. |  |  |  |
| 25 |  |  | 199 days | Death; animal was found dead by the owner. |  |  |  |

^1^VEDDRA = veterinary dictionary for drug Regulatory Activities (according to: Combined VeDDRA list of clinical terms for reporting suspected adverse reactions in animals and humans to veterinary medicinal products EMA/CVMP/PhVWP/10418/2009-Rev.11-corrigendum Page 2/264 (EudraVigilance Veterinary implementation version 16)

^2^EMEA. (2004) Guideline on harmonising the approach to causality assessment for adverse reactions to veterinary medicinal products. EMEA/CVMP/552/03-FINAL. European Medicines Agency (<https://www.ema.europa.eu>)
